# Increased Temporal Variability of Gait in ASD: A Motion Capture and Machine Learning Analysis

**DOI:** 10.3390/biology14070832

**Published:** 2025-07-08

**Authors:** Katharine Goldthorp, Benn Henderson, Pratheepan Yogarajah, Bryan Gardiner, Thomas Martin McGinnity, Brad Nicholas, Dawn C. Wimpory

**Affiliations:** 1 School of Psychology and Sports Science, Bangor University, Bangor LL57 2DG, UK; oux20a@bangor.ac.uk (K.G.); or dawn.wimpory@wales.nhs.uk (D.C.W.); 2 School of Computing, Engineering and Intelligent Systems, Ulster University, Derry (Londonderry) BT48 7JL, UKp.yogarajah@ulster.ac.uk (P.Y.); b.gardiner@ulster.ac.uk (B.G.); tm.mcginnity@ulster.ac.uk (T.M.M.); 3 Child Development Centre, Betsi Cadwaladr University Health Board (NHS), Bangor LL57 2EG, UK

**Keywords:** ASD, gait, machine learning, timing, variability

## Abstract

Motor deficits, including atypical gait, are common in individuals with autism spectrum disorder (ASD), although the precise nature and cause of this co-occurrence is unclear. Because walking is a natural activity and gait timing is a metric that is relatively accessible to measurement, we explored whether autistic gait could be described solely in terms of the timing of gait parameters. The aim was to establish whether temporal analysis, including machine learning models, could be used as a group classifier between ASD and typically developing (TD) individuals. Thus, we performed a high-resolution temporal analysis of gait on two age-matched groups of male participants: one group with high-functioning ASD and a comparison TD group (each *N* = 16, age range 7 to 35 years). The primary data were collected using a VICON^®^ 3D motion analysis system. Significant increased temporal variability of all gait parameters tested was observed for the ASD group compared to the TD group (*p* < 0.001). Further machine learning analysis showed that the temporal variability of gait could be used as a group classifier for ASD. Of the twelve models tested, the best-fitting model type was random forest. The temporal analysis of gait with machine learning algorithms may be useful as a future ASD diagnostic aid.

## Simple Summary

Research suggests that autistic peoples’ walking style may be subtly different to that of typically developing people. These differences have been shown by using advanced movement analysis called gait analysis; they cannot be seen by just watching someone walking. Previous studies, however, have produced conflicting results, perhaps because of their diverse methods and often complex approaches. We set out to test if two groups of people, one group with autism and the other a typically developing group, could be distinguished simply in terms of the micro-timing of their walking rhythm and, if an artificial intelligence technique, called machine learning, could be trained to make this classification. We found that the autistic group’s walking rhythm was clearly more variable, but on average not faster or slower, and that machine learning algorithms, trained on gait timing alone, could be used for group classification. Further validation of gait timing variability in autism is encouraged, possibly leading to a semi-automated test to assist clinicians in the early stages of their assessments, and to a fuller understanding of the nature of autism. Tests that facilitate diagnosis could lead to families being offered help sooner.

## 1. Introduction

Autism is a neurodevelopmental disorder characterised by persistent problems with social interaction, social communication, and stereotyped behaviours. Children with autism show a wide range of intellectual functioning and affected status, giving rise to the concept of an autism spectrum and the diagnostic category of autism spectrum disorder (ASD) where diagnosis is based on the criteria listed in, for example, the Diagnostic and Statistical Manual of Mental Disorders [1].

Prevalence estimates for ASD vary between geographical regions, and have generally increased over time [2,3]. In North America, a large-scale survey estimates the prevalence of ASD in 8-year-old children as 1 in 59 [4]. In addition to the core features of autism, conditions such as poor sleep and motor deficit commonly co-occur with the disorder, and there is increasing evidence for the inclusion of such phenomena in the definition of ASD [5,6]. The results of movement studies in ASD are somewhat conflicting in detail but overall support the assertion that motor deficit is common in ASD [5,7] which may not be due to developmental delay specifically [8] or, for atypical gait measures, to comorbid learning difficulty [9,10,11].

Previous research [12,13] has suggested a direct role for motor deficit in ASD by impeding the temporally and spatially coordinated execution of the motor aspects of social communication at critical stages in infant development. This reflects aspects of the hypotheses on autism aetiology that place motor anomalies in the context of a timing deficit, as suggested by some authors [14,15,16].

Previous studies have considered average step duration, cadence, or irregularities in step length or stride length patterns [17,18,19]. Several studies have reported that step length is significantly reduced in children with ASD [20,21,22,23,24], whereas others have found no such difference [17,25,26,27,28], highlighting the inconsistencies in the current findings.

These contradictory conclusions may be due, in part, to differing methodologies, small sample sizes, various inclusion criteria, and a lack of temporal resolution.

The analysis of gait in autism initially focused on indicating which brain circuits might be implicated in the disorder by comparing the gait characteristics of individuals with autism to those with disorders of known aetiology, such as Parkinson’s disease, or to those suffering the effects of cerebellar lesions that might reflect a cerebellar hypoplastic pathology [23,29] and, specifically, the loss of Purkinje neurons found in autism pathology investigations [30,31,32]. Alteration to normal gait has been shown to precede the appearance of cognitive decline in the elderly [33], in individuals genetically predisposed to Parkinson’s disease [34], and in children later diagnosed with ASD [35], suggesting a possible role for gait analysis in early detection.

Machine learning has been used to identify early gait signatures in Parkinson’s disease [36,37,38] and similar approaches for ASD-identification from gait patterns are being explored. Research has shown that the neural Network (NN) and support vector machine (SVM) classifiers were suitable for identifying the parameters of autistic gait, demonstrating that subjects can be identified as autistic based on their gait cycle at an accuracy rate of 85%, using a combination of temporal, spatial, and kinematic data [39]. A small number of studies have investigated the use of random forest (RF), decision tree (DT), Perceptron, support vector machine (SVM), k-nearest neighbor (KNN), linear discriminant analysis (LDA), and quadratic discriminant analysis (QDA) models, and a Naïve Bayes model, using kinetic and kinematic gait features for ASD classification [40,41].

The objective of this research was to focus specifically on the temporal aspects of gait, the rhythm of walking, to see if gait timing was different in ASD compared to typically developing participants, and if gait timing alone could be used as a group identifier. We sought to provide evidence, for or against, the timing deficit in ASD. We aimed to ascertain whether machine learning models could be utilised to distinguish ASD, focusing on temporal variability as a feature.

The widely used VICON^®^ system [42], with a temporal resolution of 250 Hz, was employed to measure four components of the gait cycle in the participants, including toe off to foot strike (swing duration), foot strike to toe off (stance duration), one foot strike to the next foot strike (step duration), and the strike of one foot to the next strike of the same foot (stride duration).

We hypothesised that individuals with ASD would show differences in their gait timing compared to typically developing individuals, that gait abnormality would correlate with the degree of autism symptom severity (as measured by the Autism Diagnostic Observation Schedule (ADOS) [43]), and that a machine learning analysis of the temporal measures of gait may be used as a group classifier for individuals with ASD.

## 2. Method

### 2.1. Participants

Following the defined ethical protocol, 16 male white British participants with a diagnosis of ASD were recruited from the caseloads of their current clinicians, including general practitioners. The recruitment request asked clinicians for high-functioning individuals with ASD, to avoid a confounding comorbid learning disability (LD). An analysis of clinical records identified no genetic abnormalities, comorbid diagnoses, nor medication (see Table S1 in the Supplementary Materials). There were no participants with a low birth weight (threshold set at <2.5 kg). No information was gathered regarding socioeconomic status or educational attainment. The age of the ASD participants ranged from 8 to 35 years (*M* = 15.09, *SD* = 7.71). For clarity across the age range, there were twelve children (*M* = 11.34, *SD* = 2.41) and four adults (*M* = 26.33, *SD* = 7.16).

Sixteen age-matched typically developing (TD) male white British participants were recruited from local school and university sites by specifying a required date of birth range. These TD participants were matched within 5 months to their ASD counterparts (for child and adolescent participants). As the adults were fully physically developed, they were matched to within a couple of years. The age of the TD participants ranged from 8 to 33 years (*M* = 15.03, *SD* = 7.47), including twelve children (*M* = 11.25, *SD* = 2.51) and four adults (*M* = 26.38, *SD* = 5.19). An explanation of the purpose and procedure of the experiment was given to the participants and their parents/carers as part of the recruitment requests, and full right to withdraw from the study was made explicit. The ASD participants aged 18 years or above were mental capacity assessed. No risks were identified for the participants.

All participants in the ASD group (*N* = 16) had received a clinical diagnosis of ASD from qualified and experienced clinical psychologist(s). For research purposes, we used the ADOS assessments as a metric for autism severity wherever possible. The referring clinical psychologists had conducted the ADOS assessments for 10 of the 16 ASD participants. A research psychologist provided an ADOS for a further 4 of the ASD participants. The 2 remaining participants declined to undergo further psychological assessment. The research psychologist’s reliability in the ADOS was externally assessed as 88%, and one of the referring clinical psychologists was an internationally validated ADOS trainer.

Of these *N* = 14 ADOS scores, *n* = 9 ASD participants met the threshold for autism and *n* = 5 ASD participants met the threshold for autism spectrum disorder, using the ADOS diagnostic classification. The ADOS total score combines ‘Communication’ plus ‘Reciprocal Social Interaction’ subscores, each of which were used for the comparison with gait characteristics (see Table S2 in the Supplementary Materials).

### 2.2. Informed Consent

Informed consent was obtained from all participants included in this study, or their parents, as appropriate. A professional learning disability nurse from the local government health service provider was employed to assist with the ethics protocol, recruitment, the mental capacity assessments, any relevant information sought from the medical records, seeking informed consent, plus the gait laboratory sessions involving the participants with ASD. All participants under the age of 18 were given the option of signing a consent form and received an age and capacity-appropriate information sheet. Informed consent was also sought from their parents/guardians. All information sheets for the adult participants were carefully constructed to ensure that accessibility and mental capacity assessments were conducted. The participants/parents signed informed consent waivers regarding subsequent publication of their data and were aware that this would be unidentifiable.

### 2.3. Community Involvement Statement

Numerous professionals were consulted when developing the methodology (please see the Acknowledgements). However, there were not any community members involved in the development of the present research. The motivation for the research stems from clinicians working closely with autistic people and their families, advocating that earlier diagnosis can often mean that therapeutic services are offered sooner, during a crucial window of development where strategies to facilitate social and communication skills are most helpful. A wider repertoire of diagnostic tools that can help discern autistic presentation from other developmental delays relating to social or communication could be of benefit.

### 2.4. Equipment

A 3D VICON^®^ Motion Capture System with VICON^®^ Nexus software (version 1.4.115) was used for the gait analysis. The system incorporated 12 MXF40 cameras (Vicon Motion Systems Ltd., Oxford, UK) sampling every 4 milliseconds (250 Hz) over a 4 × 4 m range and tracking 20 markers fixed to expert-informed specific points on each of the participants with specialist double-sided adhesive tape. VICON^®^ systems are widely used and commonly considered to be the gold standard for gait analysis [42].

### 2.5. Procedure

Participant height and weight were recorded via the VICON^®^ Nexus software and markers were placed on the left and right side for each of the following anatomical locations, including anterior superior iliac spine, posterior superior iliac spine, thigh, knee (medial and lateral epicondyle of the femur), tibia, ankle (fibula apex of lateral malleolus and tibia apex of medial malleolus), heel (calcaneus), and toe (head of second metatarsus) (see Figure S1 in the Supplementary Materials).

Motion capture took place in a dedicated gait analysis laboratory. The participants were asked to walk across the floor of the laboratory from corner to corner, for 6 m, while the VICON^®^ camera system made video recordings of their walks. These walks, that we called gait trials, captured the timing of multiple consecutive steps, mid-walk, once the walking rhythm was established and before deceleration. Each gait trial consisted of between 6 and 8 steps. A minimum of 5 gait trials were gathered per participant, thereby obtaining approximately 30 examples of step timing per participant. Participants were requested to repeat the gait trials until enough suitable examples were recorded. Real time quality control of the captured data excluded certain gait trials, for example, if the participant walked in a curved path, paused during the walking, or broke into a jog.

The Nexus computer software was subsequently used in a labelling process, to generate annotated stick leg computer graphic representations of the selected consecutive 6–8 steps. These graphical representations were marked at the anatomical points corresponding to the reflective markers attached to the participants. Following this labelling process, the time point of every left foot strike, right foot strike, left toe-off and right toe-off, etc. was recorded for each participant, by moving forward, frame-by-frame, through the video and recording the corresponding frame number (time point at a temporal resolution of 250 Hz) in relation to the time course of the gait trial.

Recorded temporal gait parameters included step duration (from one foot striking the floor to the opposite foot striking the floor), swing duration (from toe-off of one foot to foot strike of the same foot), stance duration (from foot strike to toe off, of the same foot), and stride duration (from one foot striking the floor to the same foot striking the floor). This temporal data enabled the calculation of mean, median, standard deviation, and coefficient of variation (CoV) for each gait parameter. The mean and CoV values were then taken as the dependent variables of a between-subjects design, comparing the quasi-independent variables and the ASD subjects with the age-matched TD comparison group.

### 2.6. Statistics and Machine Learning

Kolmogorov–Smirnov and Levene tests were conducted for all tests to assess normality and homogeneity of variance, respectively. If these parametric criteria were satisfied, then independent measures t-tests were run, using age-matched subjects in a between-subjects design. If non-parametric tests were required, Mann–Whitney U tests were run with the effect size (SPSS Statistics 27).

The results of the statistical tests were used to determine the gait features to be used for the training of 12 different machine learning model types, including random forest (RF), decision tree (DT), perceptron, support vector machine (SVM), logistic regression (LR), k-nearest neighbor (KNN), bagging, AdaBoost, Gaussian Naïve Bayes (GNB), gradient boosting, linear discriminant analysis (LDA), and quadratic discriminant analysis (QDA), with the goal of automatically differentiating between individuals with ASD and the TD comparison group. For each model type, two models were trained, one using only the features that were found to be significantly different for the ASD and TD groups (Feature Set A), and one using all available gait features (Feature Set B). Due to the initial breakdown of the dataset representing each individual as a single feature vector (Subset A), there are only 32 inputs from which the machine learning models can train.

There is evidence that suggests a link exists between predictor performance and dataset size [44]; therefore, a second representation from the same initial dataset was considered for training that increases the number of training inputs (Subset B). Instead of taking the mean and coefficient of variance of each feature for each subject, the mean and coefficient of variance was calculated for every gait trial for every individual. The same analytic tests were applied to Subset B to identify the significant features for training (Feature Set C). Again, all available gait features within Subset B, were also used to train sets of models for comparison (Feature Set D).

In performing this, two effects were achieved. Firstly, the number of inputs for training immediately increases from 32 to 295; secondly, imbalance was introduced into the training data (163 ASD and 132 TD inputs) due to each individual having a differing number of gait trials recorded. To deal with the introduced imbalance, a data generation algorithm, Borderline SMOTE [45], was employed such that the ratio in number of ASD and TD inputs would be closer to 1:1, while also increasing the total number of inputs further.

The Borderline SMOTE algorithm was applied in two ways, as follows: the first considers the ASD and TD groups as the two variables to be balanced (Generated A); the second considers each individual as its own group so that the number of gait trials representing each individual is what is being balanced (Generated B). Both methods result in the ratio of the number of inputs for the ASD and TD groups tending towards 1:1; however, the Generated B method generates more inputs than the Generated A method (A: 163 ASD and 163 TD vs. B: 256 ASD and 245 TD).

To train the models, a strategy combining 12 normalisation, 10-fold cross validation, and a grid-based parameter search was employed. The resulting accuracy, using the best set of parameters and the mean cross validation accuracy values, was then used to compare each of the models. In total, 6 sets of models were trained using different combinations of how the data was represented, which features were used, and which data generation technique was applied (see Table S3 in the Supplementary Materials).

## 3. Results

### 3.1. Per-Subject and Per-Trial Analysis

The results were analysed, both on a per-subject and a per-trial basis for each phase of the gait cycle to include step, stance, swing, and stride, and further resolved as ‘Left’ and ‘Right’ subcategories—creating eight gait features of interest.

The first set of results details the statistical analysis of the ASD group (*N* = 16) vs. the TD group (*N* = 16) on a per-subject basis. The data of each of the ASD participants and the TD comparison group produced 12 coefficient of variation (CoV) values and 12 mean values per participant. The per-subject analysis was conducted using all appropriate walking trials (180 ASD trials and 146 TD trials).

The second set of results details the statistical analysis of between-group differences for the combined walking trials from all *N* = 16 ASD participants and the combined walking trials from all *N* = 16 TD participants. A pruning process was applied before the data was analysed. Gait cycles with any missing values and gait trials with less than two complete gait cycles, or without a recorded trial time, were removed. This was required to keep the format of the data consistent with how it was used during the machine learning stage. After pruning, a total of 163 gait trials for the ASD and 132 gait trials for the TD groups were compared.

### 3.2. Gait Variability—‘Per-Subject’ Analysis

The per-subject analysis compared the 16 ASD participants (*N* = 180 trials total) and the 16 TD age-matched participants (*N* = 146 trials total). Each of the 32 participants had eight coefficient of variation (CoV) timing values encompassing step, stance, swing, and stride parameters. These were calculated per-subject from all suitable examples of their gait cycles, using the formula CoV = standard deviation/mean. There was an average of 11.25 suitable walking trials for the ASD participants and 9.13 for the TD comparison group. The number of examples of each phase of the gait cycle varied between the participants; for instance, the average number of examples for each phase of the gait cycle ranged from 34.29 to 39.94, across the ASD participants (overall average of 37.15).

Normality and homogeneity of variance assumptions were not satisfied, so Mann–Whitney U tests were employed (see Table S4 in the Supplementary Materials). A one-tailed null hypothesis predicted equal variability in the timing of gait in the ASD group to that of the TD comparison group.

**Figure 1:**
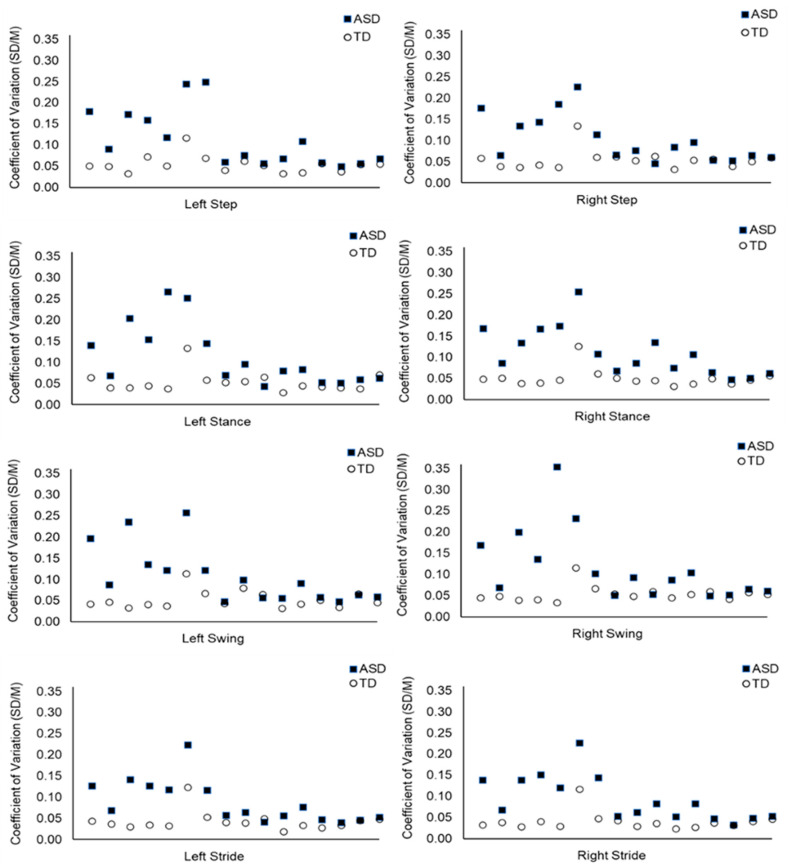
Temporal Variability Within the Gait Cycle: Mean CoV Values Per Subject for the Duration of the Step, Stance, Swing, and Stride Phases. Note: Each data point represents the CoV value of one participant (*n* = 32). The ASD participants are displayed at the same point on the *x* axis as their age-matched TD participant. Step, stance, swing, and stride CoV values are calculated using the duration of time taken to execute these aspects of the gait cycle.

The null hypothesis was rejected. The temporality of the gait cycle was significantly more variable in the ASD compared to the TD group. For example, there was more temporal variability within the left stride in the ASD (*Mdn* = 0.066) than TD group (*Mdn* = 0.038), *U* = 227, *z* = 3.73, *p* < 0.001; see Table 1. The right stride also showed greater temporal variability for the ASD (*Mdn* = 0.075) than the TD group (*Mdn* = 0.037), *U* = 236, *z* = 4.07, *p* < 0.001. The temporality of gait was significantly more variable for the ASD than the TD group for step, stance, swing, and stride parameters. See Table 1 and Figure 1 below (plus see Figure S2 in the Supplementary Materials).

### 3.3. Average Gait Timing—‘Per-Subject’ Analysis

Each participant also had eight mean timing values for step, stance, swing, and stride (left and right separately), calculated from all examples within their multiple trials. Normality and homogeneity of variance assumptions were satisfied for step, stance, and stride data for average timing, so *t* tests were employed. The swing data did not satisfy normality and homogeneity of variance criteria, so a Mann–Whitney U test was utilised. A two-tailed hypothesis predicted no difference in the average timing of gait parameters in the ASD group compared to the TD group. The null hypothesis was accepted, with no significant difference was found between the average duration of gait parameters for the ASD subjects compared with the TD group. See Table 2 below.

### 3.4. Gait Variability—‘Per-Trial’ Analysis

The data was analysed on a per-trial basis between the ASD and the TD participants. The same aspects of the gait cycle were investigated, including step, stance, swing, and stride, and each were further subdivided into the left and the right—providing eight (CoV) timing factors to compare between the ASD (*N* = 16) and the TD (*N* = 16) groups. The CoV values were calculated for each trial, rather than presenting a CoV value per participant. This compared a total of 163 gait trials for the ASD group and 132 gait trials for the TD group. There was an average of 10.19 examples of gait trials for the ASD participants and an average of 8.88 examples of gait trials for the TD group.

Normality assumptions were assessed using the Kolmogorov–Smirnov test and homogeneity of variance assumptions were assessed using the Levene test. These assumptions were not satisfied for any analyses, so Mann–Whitney U tests were employed throughout. A one-tailed hypothesis predicted more variability in the timing of gait of the ASD group than the TD group. The null hypothesis was rejected. The temporality of the gait cycle was significantly more variable in the ASD (*Mdn* = 0.028) than the TD (*Mdn* = 0.020) participants for the left stride, *U* = 14,176.0, *z* = 4.69, *p* < 0.001, *r* = −0.32. The temporality of the gait cycle was significantly more variable during the right stride in the ASD (*Mdn* = 0.026) than the TD group (*Mdn* = 0.017), *U* = 14,295.0, *z* = 4.86, *p* < 0.001, *r* = 0.33. There was increased temporal variability for all eight aspects of the gait cycle, in the ASD group. See Table 3 below.

### 3.5. Average Gait Timing—‘Per-Trial’ Analysis

In this analysis, each participant also had eight mean timing values, one for every phase of the gait cycle, including step, stance, swing, and stride (left and right separately). These were calculated from all *N* = 163 gait trials combined for 16 ASD participants compared to all *N* = 132 gait trials combined for the 16 TD participants. In contrast to the variability, it was predicted that there would be no difference between the ASD and the TD groups in their average timing for the four elements of the gait cycle. Normality and homogeneity of variance assumptions were satisfied for the right step phase only. An independent t-test was therefore used for the right step, and Mann–Whitney U tests were employed for all other analyses.

There were no significant differences between the ASD and TD participant’s average gait timings using the per-trial analysis method. See Table 4 below.

### 3.6. ADOS and Gait Variability

The severity of the ASD symptoms was also considered in relation to the CoV data for each test. An analysis was conducted using the ADOS Communication (C) scores, the ADOS Reciprocal Social Interaction (RSI) scores, and the ADOS Total scores (C + RSI). A correlative analysis was run using Spearman’s rho to establish any relationship between the per-subject CoV of step, swing, stance, and stride and the ADOS scores. There was no correlation found between step, stance, swing, or stride variability and the ADOS scores for the *N* = 14 ASD participants with an ADOS assessment (see Table S5 in the Supplementary Materials).

### 3.7. Tests for Potential Confounds

#### 3.7.1. Gait Variability and Age

An additional correlative analysis tested for any relationship between gait timing and chronological age. Pearson’s was used to assess the relationship between CoV (per-subject analysis) and age in months, revealing no correlation between gait timing and chronological age for left step (*r* = −0.40, *n* = 16, *p* = 0.12) or right step (*r* = −0.28, *n* = 16, *p* =0.29); left stance (*r* = −0.23, *n* = 16, *p* = 0.39) or right stance (*r* = −0.40, *n* = 16, *p* = 0.13); left swing (*r* = −0.25, *n* = 16, *p* = 0.35) or right swing (*r* = −0.15, *n* = 16, *p* = 0.57); left stride (*r* = −0.35, *n* = 16, *p* = 0.19) or right stride (*r* = −0.39, *n* = 16, *p* = 0.13).

#### 3.7.2. Inter-Rater Reliability for the ADOS

Clinical ADOS assessment videos were available to the researcher for *n* = 8 participants, which were re-coded (blind) for the purpose of establishing inter-rater reliability between the researcher and the clinicians. A Spearman’s rho was run to ensure a high correlation between an ADOS certified researcher and the clinicians’ scoring (*n* = 8), with a positive significant result for the Communication score, *r* = 0.86, *p* = 0.007; the Reciprocal Social Interaction score, *r* = 0.83, *p* = 0.010; and the combined ADOS Total score, *r* = 0.96, *p* < 0.001. The value of the coefficient of determination (R^2^ = 0.92 for the ADOS Total score) implied inter-rater reliability of 92%.

#### 3.7.3. Body Mass Index

The weight percentiles for all participants were used for correlative analysis, to ascertain if there was any association between weight and the temporal variability of gait. After selecting the appropriate correlational tests, there was no association found between weight percentiles and temporal variability. See Table 5 below (plus Table S6 in the Supplementary Materials).

#### 3.7.4. Machine Learning Analysis—Feature Set Results

The feature sets used for training were produced using the results from the statistical tests (see methodology). Feature Sets A and B were derived from the per-subject analysis and Feature Set C and D were derived from the per-gait trial analysis. The only caveat to this is the CoV for cadence when using Subset B. As cadence only has one value per trial, the CoV for cadence will always be 0, and is therefore not included in Feature Sets C and D. Models are reported using the accuracy with the best parameters (accuracy) and the mean cross validation accuracy (mean cv accuracy). The combination of features included in each feature set is summarised in Table 6 below.

The best model accuracy and mean cv accuracy achieved in each test are reported in Table 7 below (further details can be found in Tables S7–S12 in the Supplementary Materials).

Test 1 and Test 2 varied only by the feature set used, with Test 1 using Feature Set A and Test 2 using Feature set B. The results show that this change did not achieve any difference in the best model accuracy; however, there was a large increase of 31.67% in the mean cross validation accuracy. The absence of an improvement on the best accuracy when including all features shows the effectiveness of the feature selection methods used. Achieving a higher mean cv accuracy in Test 2 compared to Test 1, however, implies that the features chosen for Feature Set A could be overfitting to the available data.

The use of Subset B in Test 3, in combination with data generation using the Generation A method, was able to achieve more consistent results across all models than in Tests 1 and 2, with a total range of accuracies reported being 18.84%, compared to Test 1 and 2’s range of 71.43%. This advantage came at the cost of the maximum accuracy achieved dropping to 63.77%. One factor that could affect this is the difference in the amount of data available for training and testing between the two subsets of data. These results show that the higher variation per subject captured in Subset B provides a more complete representation of gait examples that can occur in real world scenarios. This leads to more consistent results, while also introducing more noise that can increase the complexity of the ASD classification challenge, resulting in less accurate models.

Introducing the data generation technique labelled Generation B, replacing Generation A, Test 4 obtains higher mean cv accuracy values, closer to those obtained in Tests 1 and 2. Test 4 also retained the consistency across the models that was achieved by using Subset B over Subset A in Test 3. This can be attributed to each individual now being represented equally in the dataset, while also keeping the balance between the amount of ASD and TD data. Test 5 can also be compared to Test 3, with the difference here being the use of Feature Set D in Test 5 over Feature Set C. In comparison with Test 3, Test 5 was also able to increase the accuracy achieved by the best model and retain consistency in the results, although to a lesser degree than seen between Test 3 and Test 4. This implies that, although the feature selection process deemed some features to not be significant for the detecting autism, they still hold some useful information for this purpose when applied to a larger dataset that includes more variation. Considering the results from comparing the previous tests, it is no surprise that Test 6, which combined each of the methods that produced improved accuracies and consistency, achieved the best overall mean cv accuracy and a relatively high best model accuracy. The model type which achieved this was the random forest, which also had the best performance in Test 5 and performed well in most of the previous tests. This suggests that it is well suited to ASD classification using temporal gait features. The improvements were not isolated to the random forest model in Test 6, with the decision tree, KNN, bagging, gradient boosting, and QDA models also achieving relatively high accuracies (>75%) when compared to previous performances. This shows that the use of the per-trial representation, alongside generating synthetic data per subject in combination with using all calculated features is the optimal configuration for the ASD classification using this dataset.

## 4. Discussion

### 4.1. Overview: Gait Analysis Results

This research describes a high-resolution analysis of temporal aspects of gait in ASD to investigate the possibility of ASD-associated timing differences and to determine whether ASD and TD individuals can be distinguished based on gait timing alone. Along with standard statistical analyses, the investigation included the application of artificial intelligence in the form of machine learning algorithms.

The statistical analysis showed significantly increased temporal variability of step, swing, stance, and stride duration for the children with ASD, compared with the TD subjects, *p* < 0.001. The results suggest that analysis of a single parameter, timing, divorced from any of the other complex gait measures, such as force or joint angle, is sufficient for group discrimination between the ASD and TD cohorts.

Interestingly, no significant differences for the average timing of step, swing, stance, and stride duration were observed between the groups. There was also no relationship between the ADOS assessment test scores and gait variability, tentatively suggesting that temporal variability could be present regardless of the ‘severity’ of autism. However, a comorbid diagnosis of learning disability was an exclusionary criterion for recruitment, and so the ADOS scores might not have represented a wide enough range to demonstrate a relationship between gait variability and the ADOS score.

### 4.2. Machine Learning and Gait Analysis

The existing literature had previously investigated the use of temporal, kinetic, and kinematic gait features in training machine learning models for the purpose of ASD classification [39,40,41,46,47]. This study expands on the existing literature in the application of gait data to the ASD classification problem in three main areas. Firstly, the current study includes the variance within the temporal data as a specific set of features when training machine learning models. The models trained only on variance-based features suffered from overfitting, shown by a low mean cv accuracy of 45%, while holding a best accuracy of 85.71%. This issue was overcome by using a combination of the variance-based and averaged temporal features, achieving a mean cv accuracy of 76.67% by comparison. This set of features were then used to achieve the best overall model with an 82.18% accuracy and an 82.00% mean cv accuracy. Secondly, a set of 12 different classifier types were trained across multiple tests, allowing for better comparison and a more informed classifier choice for future studies. It was found that the RF classifier performed consistently well across multiple tests while also obtaining the best overall model. Finally, the current study applied the Borderline SMOTE algorithm as a method of data generation to handle class imbalance. Its effect was tested on model accuracy when applied at class and subject level. Applying the Borderline SMOTE algorithm at the subject level improved the consistency of results across all models and was used to achieve the best overall model.

### 4.3. Neurological Implications of Temporal Variability in Gait

In healthy adults, the temporal variability of gait is characteristically low, less than a few percent [48] compared with the more variable pattern of childhood [49,50]. By contrast, increasing gait variability is associated with the onset of neurodegenerative disorders, such as Parkinson’s disease and Alzheimer’s disease, but notably, the increased temporal variability of gait is not associated with old age per se [51]. Thus, temporal regulation of the gait cycle with its constrained variability is a characteristic of normal neural function, and increased variability may indicate neurological impairment or an abnormal developmental trajectory.

Increased variability in gait parameters has previously been reported in ASD [26,27,28,52]. As gait variability is a characteristic of Parkinson’s disease and cerebellar ataxia [53,54,55] (conditions that involve the basal ganglia and cerebellum, respectively), previous research has suggested that these brain regions may be implicated in ASD [28,29,56,57,58,59,60,61]. Under certain conditions, variability in stride time can be influenced by cognitive load, general physiology, and mental health [53,62,63]. Thus, our experimental design excluded individuals with learning disability and ensured that the subjects walked at their natural pace, in their usual way.

Loss of dopaminergic neurons within the basal ganglia circuitry is the primary cause of Parkinson’s disease [64], and this circuit has been implicated in ASD clinically [56,58], by voxel-based morphometric analyses [65], and by functional-MRI [66,67,68,69]. However, although certain aspects of ASD are perhaps explainable in terms of basal ganglia pathology (particularly repetitive behaviour and motivational effects), how the basal ganglia pathology could relate to cardinal aspects of ASD, such as profound problems with sociability and communication, is unclear.

Increased temporal variability of elements of the gait cycle is also found in individuals with cerebellar ataxia and cerebellar lesions, and there is a long history of association of cerebellar anomaly with ASD [70,71,72,73,74]. However, only recently has progress begun to be made as to how such cerebellar pathology could impact the core features of autism. Interestingly, an ASD mouse model where the TSC complex subunit 1 (*Tsc1*) gene was deleted solely in the Purkinje cells of the cerebellum showed social deficits and repetitive/inflexible behaviours due to the disruption of functional connectivity between the medial prefrontal cortex and the cerebellum. Furthermore, this circuit was found to be disrupted in other genetic mouse models of ASD [75,76,77]. In addition, the ASD associated gene *RORA* [78], an essential gene for normal development of cerebellar Purkinje neurons can harbour loss-of-function variants causative of LD with autism in humans that might represent a point of both genetic and environmental vulnerability for ASD [79,80,81].

## 5. Applications

This research indicates that the temporal variability of gait is a distinguishing characteristic of ASD that is independent of age. Considerable human resources are required for ASD diagnosis involving clinical behavioural assessments using psychological instruments and expert clinical opinion. Earlier diagnosis and intervention leads to improved outcomes. To this end, a semi-automated temporal variability assessment of gait could be a possible diagnostic support for clinicians, increasing the efficiency of diagnostic services for the burgeoning numbers of children and adults currently seeking ASD diagnostic consultations. Resource-wise, access to gait analysis equipment may require an initial cost for health services, but we anticipate that, if gait timing is validated as an ASD metric, it would facilitate diverse possibilities for timing data collection. The complex camera and force plate arrangements that are required for data capture of joint angle etc. for conventional gait analysis may not be necessary for one dimensional timing data. Concomitantly, less complex and less expensive signal transduction devices, e.g., microphones may be sufficient to capture timing data sonically, for example.

Understandably, gait analysis would only be appropriate for physically mobile individuals without a physical disability, so, on a practical level, it would not be accessible to every patient. However, the majority of individuals on a neurodevelopmental waiting list are able to walk effectively.

In terms of age range, this participant cohort spanned early childhood to adulthood, indicating that gait analysis methods could be a benefit to a wide range of society.

## 6. Limitations

This research recruited only a small sample, thus, although the positive results must be interpreted cautiously, they do, however, encourage further investigation in a larger sample. Only temporal gait parameters were acquired, while typical gait research includes additional kinetic and kinematic parameters. However, our intention was not to replicate previous findings, but to focus on the temporal variability of gait in ASD specifically and whether temporal measures alone were sufficient to allow for group classification. We anticipate that further work might explore the specificity of the observed increased variability in ASD by comparisons with other neurodivergent groups, something that was beyond the scope of this pilot study.

The typically developing participants were matched with participants with ASD by age, but features such as physical activity level and socioeconomic status, which could have some influence on gait, were not recorded for either group. Metrics of cognitive ability existed for the ASD group, but were not obtained for the typically developing group, although all TD individuals were in/had received mainstream school education.

## 7. Conclusions

This research focused specifically on the temporal aspects of gait to see if gait timing was different in ASD participants compared to typically developing participants, and if machine learning algorithms trained on gait timing alone could be used as a group classifier. We found that the temporal variability of gait for a group of individuals with ASD was significantly increased compared to an age-matched typically developing group, and that machine learning models could effectively distinguish ASD using temporal variability as a feature. The results supported the hypothesis that individuals with ASD exhibit temporal anomalies in their gait cycle, and the findings encourage replication in a larger sample. In the future, measurement of the temporal variability of gait, in conjunction with machine learning algorithms, may contribute to motor tests to assist ASD diagnostic processes.

## Figures and Tables

**Table 1:** Temporal variability within the gait cycle-per-subject analysis.

Phase of Gait Cycle		Coefficient of Variation (CoV) = Standard Deviation/Mean (0.000)				Mann–Whitney *U* Results				CoV (%) = (SD/M) × 100	
		Mean ASD	Mean TD	Mdn ASD	Mdn TD	*U*	*z*	*p*	*r*	Mean ASD	Mean TD
Step	L	0.113	0.054	0.084	0.052	218	3.39	<0.001	0.60	11.35	5.43
	R	0.103	0.055	0.081	0.053	220	3.47	<0.001	0.61	10.30	5.48
Stance	L	0.115	0.054	0.082	0.045	219	3.43	<0.001	0.61	11.47	5.37
	R	0.112	0.051	0.097	0.047	237	4.11	<0.001	0.73	11.20	5.11
Swing	L	0.109	0.052	0.089	0.044	216	3.32	<0.001	0.59	10.85	5.25
	R	0.117	0.054	0.090	0.050	216	3.32	<0.001	0.59	11.72	5.39
Stride	L	0.088	0.043	0.066	0.038	227	3.73	<0.001	0.66	8.76	4.30
	R	0.094	0.041	0.075	0.037	236	4.07	<0.001	0.72	9.38	4.05

Note. Mean values were calculated from raw temporal data sampled at 250 Hz. Abbreviations: L = left; R = right; ASD = autism spectrum disorder; TD = typically developing; “*U*” represents the test statistic; “*z*” is the standardized test statistic; “*p*” is the *p*-value; and “*r*” is the effect size.

**Table 2:** Average gait timing-per-subject analysis.

Phase of Gait Cycle		Mean Values Calculated from Raw Temporal Data Sampled at 250 Hz				Independent Samples *t* Test		Seconds (Raw Values × 0.004)	
		Mean ASD	Mean TD	SEM ASD	SEM TD	*t* (30)	*p*	Mean ASD	Mean TD
Step	L	133.75	127.93	3.59	3.88	−1.100	0.28	0.535	0.512
	R	130.73	126.36	3.59	3.90	−0.825	0.42	0.523	0.505
Stance	L	149.62	144.70	4.51	4.93	−0.736	0.47	0.598	0.579
	R	151.00	145.04	4.43	4.85	−0.908	0.37	0.604	0.580
Stride	L	264.02	254.18	7.18	7.79	−0.929	0.36	1.056	1.017
	R	264.21	254.63	6.75	7.75	−0.932	0.36	1.057	1.019
		Mean ASD	Mean TD	SEM ASD	SEM TD	Mann–Whitney		Mean ASD	Mean TD
						*U*	*p*		
Swing	L	114.98	109.65	5.45	2.96	135	0.81	0.460	0.439
	R	120.08	109.48	7.61	3.07	146	0.52	0.480	0.438

Note. Mean values were calculated from raw temporal data sampled at 250 Hz. L = left; R = right; *p* value two-tailed. The test statistic is “*t*”, using degrees of freedom = 30; or “*U*” according to test used; *p* = *p*-value.

**Table 3:** Temporal variability within the gait cycle-per-trial analysis.

Phase of Gait Cycle		Coefficient of Variation (CoV) = Standard Deviation/Mean (0.000)				Mann–Whitney U Results				CoV (%) = (SD/M) × 100	
		Mean ASD	Mean TD	Mdn ASD	Mdn TD	*U*	*z*	*p*	*r*	Mean ASD	Mean TD
Step	L	0.056	0.031	0.041	0.028	14,428.5	5.04	<0.001	0.34	5.56	3.06
	R	0.056	0.034	0.044	0.031	14,180.0	4.70	<0.001	0.32	5.65	3.37
Stance	L	0.053	0.030	0.036	0.027	13,837.5	4.23	<0.001	0.29	5.28	2.95
	R	0.046	0.027	0.034	0.025	13,801.5	4.18	<0.001	0.28	4.61	2.66
Swing	L	0.058	0.030	0.042	0.026	15,087.0	5.94	<0.001	0.40	5.80	2.98
	R	0.055	0.034	0.044	0.031	14,146.5	4.65	<0.001	0.31	5.55	3.41
Stride	L	0.037	0.021	0.028	0.020	14,176.0	4.69	<0.001	0.32	3.69	2.10
	R	0.039	0.019	0.026	0.017	14,295.0	4.86	<0.001	0.33	3.93	1.86

Note: Mean values were calculated from raw temporal data sampled at 250 Hz. Abbreviations: L = left; R = right; ASD = autism spectrum disorder; TD = typically developing; “*U*” represents the test statistic; “*z*” is the standardized test statistic; “*p*” is the *p*-value; and “*r*” is the effect size.

**Table 4:** Average Gait Timing-Per-Trial Analysis.

Phase of Gait Cycle		Mean Values Calculated from Raw Temporal Data Sampled at 250 Hz				Mann–Whitney U Results			Seconds (Raw values × 0.004)	
		Mean ASD	Mean TD	Mdn ASD	Mdn TD	*U*	*z*	*p*	Mean ASD	Mean TD
Step	L	132.89	128.77	133.75	133.3	11,798	1.43	0.15	0.53	0.52
	R	130.50	127.57	130.83	132.1	1.443 **	-	0.15	0.52	0.51
Stance	L	149.47	146.04	147.67	151.3	11,553	1.09	0.28	0.60	0.58
	R	150.70	146.12	149.33	152.5	11,755	1.37	0.17	0.60	0.58
Swing	L	113.61	110.40	111.57	112.0	11,134	0.52	0.60	0.45	0.44
	R	117.48	110.46	110.00	111.3	11,226	0.64	0.52	0.47	0.44
Stride	L	262.75	256.23	264.50	266.4	11,756	1.37	0.17	1.05	1.02
	R	263.00	256.61	265.00	267.2	11,813	1.49	0.15	1.05	1.03

Note. Mean values were calculated from raw temporal data sampled at 250 Hz. Abbreviations: L = left; R = right; ASD = autism spectrum disorder; TD = typically developing; “*U*” represents the test statistic; “*z*” is the standardized test statistic; “*p*” is the *p*-value. ** The t-statistic is reported for the right step phase where the independent t-test was used.

**Table 5:** Correlative Analysis Between Weight Percentiles and Gait Timing for *n* = 16 ASD participants: Weight Does Not Account for Variability in ASD Gait.

Phase of Gait Cycle	Kolmogorov–Smirnov		Spearman’s Rho		Pearson’s	
	D (16)	*p*	*r* (14)	*p*	*r* (14)	*p*
Left step	0.21	0.059	−0.19	0.478	−0.31	0.243
Right step	0.19	0.128	0.37	0.161	−0.43	0.101
Left stance	0.23	0.024 *	−0.38	0.147	n/a	n/a
Right stance	0.17	0.200	−0.20	0.451	−0.34	0.195
Left Swing	0.18	0.153	−0.09	0.729	−0.30	0.256
Right swing	0.25	0.009 *	−0.19	0.485	n/a	n/a
Left stride	0.21	0.055	−0.28	0.289	−0.46	0.074
Right stride	0.21	0.070	−0.24	0.368	−0.41	0.120

Note. *r* and D values given to two decimal places, *p* given to three decimal places. D (16) = Kolmogorov–Smirnov test statistic, with a sample size of 16. Spearman’s Rho correlation coefficient used throughout: small ASD-only sample; some components of gait cycle violated parametric requirements for alternative correlative tests, these are marked with * by the Kolmogorov–Smirnov statistic. Pearson’s reported only where data met the criteria for normality; where this assumption is not met “n/a” = “not applicable”. Coefficient of variation (CoV) values for each ASD participant were used as the value for gait variability. The correlation coefficient is denoted using *r*, with degrees of freedom = 14.

**Table 6:** Feature Type Combinations for Each Feature Set Used During Model Testing.

Feature Group	A	B	C	D
Mean Gait Phase Timings	No	Yes	No	Yes
CoV Phase Timings	Yes	Yes	Yes	Yes
CoV Cadence	Yes	Yes	No	No
Mean Cadence	No	Yes	No	Yes

**Table 7:** Accuracy Results for Machine Learning Models Performing ASD Classification Across all Six Tests.

Test Number	Best Model	Accuracy (%)	Mean CV Accuracy (%)
1	AdaBoost	85.71	45.00
2	Decision Tree	85.71	76.67
3	Random Forest	63.77	53.94
4	Random Forest	61.80	71.87
5	Random Forest	75.76	68.85
6	Random Forest	82.18	82.00

## Data Availability

The original contributions presented in this study are included in the article/Supplementary Material. Further inquiries can be directed to the corresponding author(s). The participants’ raw temporal gait data has been anonymised and is deposited in Figshare. https://figshare.com/articles/dataset/Increased_Temporal_Variability_of_Gait_in_ASD_A_Motion_Capture_and_Machine_Learning_Analysis/16559982 (accessed on 27 May 2025).

## Supplementary Materials

The following supporting information can be downloaded at: https://www.mdpi.com/article/10.3390/biology14070832/s1, Figure S1: Anatomical Marker Placement used for VICON Motion Capture; Figure S2: Temporal Variability Within the Gait Cycle: Per-Subject Statistical Analysis Table S1: Age and Relevant Medical History of N = 16 ASD Participants; Table S2: Autism Diagnostic Observation Schedule (ADOS) Scores for N = 14 ASD Participants; Table S3: Component Combinations that Make Up Each of the 6 Tests for Model Training; Table S4: Tests for Normality and Homogeneity of Variance - ‘Per-Subject’ Analysis; Table S5: No Relationship between Gait Variability and ADOS Assessment Scores; Table S6: Weight Percentiles of ASD Participants; Table S7: Test 1 Accuracy Results for Machine Learning Models Performing ASD Classification; Table S8: Test 2 Accuracy Results for Machine Learning Models Performing ASD Classification; Table S9: Test 3 Accuracy Results for Machine Learning Models Performing ASD Classification; Table S10: Test 4 Accuracy Results for Machine Learning Models Performing ASD Classification; Table S11: Test 5 Accuracy Results for Machine Learning Models Performing ASD Classification; Table S12: Test 6 Accuracy Results for Machine Learning Models Performing ASD Classification.
