# Supplementary material for: Increased Temporal Variability of Gait in ASD: A Motion Capture and Machine Learning Analysis
Source: Biology (Basel). 2025 Jul 8;14(7):832. doi: 10.3390/biology14070832 (PMC12292520; doi:10.3390/biology14070832)
Supplement: Supplementary file 1 [file biology-14-00832-s001.zip › biology-3696239-supplementary.pdf]

Fig. S1

Anatomical Marker Placement used for VICON Motion Capture

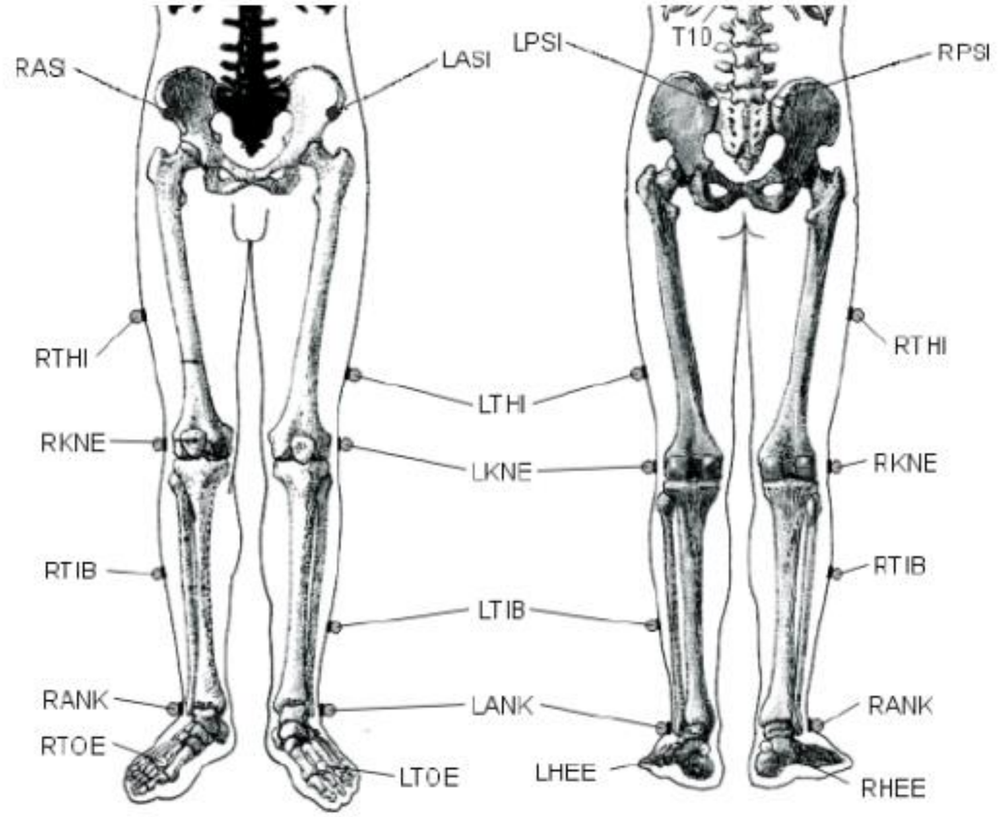

Note. As retrieved from http://www.idmil.org/mocap/Plug-in-Gait+Marker+Placement.pdf in 2013, permissions received. Abbreviations listed below:

Left and Right Anterior Superior Iliac spine = LASI and RASI

Left and Right Posterior Superior Iliac spine = LPSI and RPSI

Left and Right Thigh = LTHI and RTHI

Left and Right Knee = LKNE and RKNE

Left and Right Tibia = LTIB and RTIB

Left and Right Ankle = LANK and RANK

Left and Right Heel = LHEE and RHEE

Left and Right Toe = LTOE and RTOE

Fig. S2
Temporal Variability Within the Gait Cycle: Per-Subject Statistical Analysis

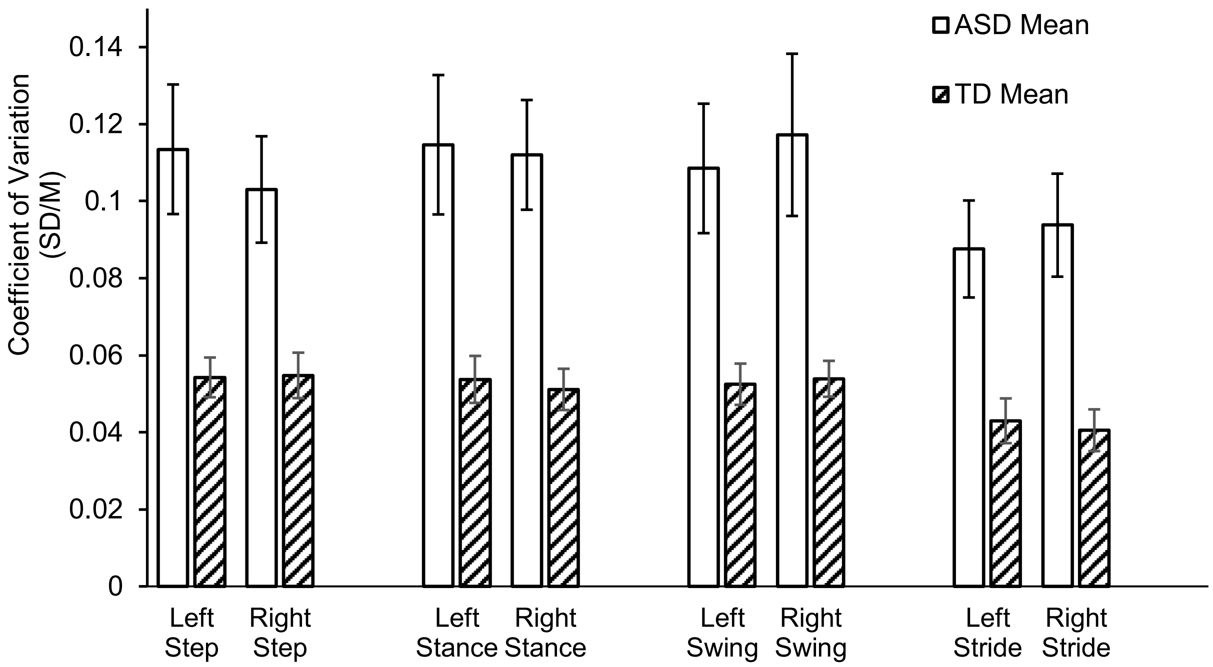

Note. Error bars shown to +/- 1 SEM

ASD Mean = Mean Coefficient of Variation values for (*N* = 16) ASD participants

TD Mean = Mean Coefficient of Variation values for (*N* = 16) TD participants

Table S1

Age and Relevant Medical History of N = 16 ASD Participants

Participant ID	Age in years	Birth weight (kg)	Genetic disorder (routine tests)	Comorbid diagnoses	Medication at time of participation
2	9.83	3.41	None	None	None
3	11.83	2.67	None	None	None
4	20.83	3.22	No record, but very high functioning		
11	12.08	2.91	None	None	None
12	14.08	4.00	None	None	None
13	8.42	2.77	None	None	None
14	8.92	4.14	None	None	None
15	10.33	3.77	None	None	None
17	12.08	3.08	None	None	None
18	14.92	4.54	None	None	None
20	29.42	2.52	No record	No record	No record
28	11.25	4.24	None	None	None
29	14.92	3.49	None	None	None
35	20.03	2.91	Less tests – no concerns		None
36	35	Unknown	No record, but very high functioning		
37	8.33	4.27	None	None	None

Table S2

Autism Diagnostic Observation Schedule (ADOS) Scores for N = 14 ASD Participants

	ADOS Scores			ADOS	
Participant ID	Communication (C)	Reciprocal Social Interaction (RSI)	Total (C + RSI)	Classification Diagnosis	Administrator and coder
2	6	12	18	Autism	Clinician
3	2	7	9	ASD	Clinician
11	4	13	17	Autism	Researcher
12	2	6	8	ASD	Clinician
13	5	11	16	Autism	Researcher
14	3	8	11	ASD	Clinician
15	5	7	12	Autism	Clinician
17	2	11	13	Autism	Clinician
18	2	11	13	Autism	Clinician
20	5	13	18	Autism	Researcher
28	5	9	14	Autism	Clinician
29	5	8	13	Autism	Clinician
36	3	4	7	ASD	Researcher
37	3	9	12	ASD	Clinician

Note. The *N* = 14 ADOS scores detailed here are the ones used in the reported analysis and consisted of *n* = 10 clinician scores and *n* = 4 researcher scores. ASD = Autism Spectrum Disorder

Table S3

Component Combinations that Make Up Each of the 6 Tests for Model Training

Test	Data Subset	Feature Set	Data Generation Method
1	A	A	None
2	A	B	None
3	B	C	A
4	B	C	B
5	B	D	A
6	B	D	B

Table S4

Tests for Normality and Homogeneity of Variance - 'Per-Subject' Analysis

		Kolmogorov-Smirnov		Shapiro-Wilk		Levene's (based on Mean)	
Phase of gait cycle	Group	*D*(16)	*p*	*W*(16)	*p*	*F*(1,30)	*p*
L Step CoV	TD	0.205	0.071	0.820	0.005	18.462	0.000
	ASD	0.210	0.059	0.833	0.008		
R Step CoV	TD	0.298	0.000	0.685	0.000	13.482	0.001
	ASD	0.190	0.128	0.870	0.027		
L Step Mean	TD	0.158	0.200	0.961	0.689	0.742	0.396
	ASD	0.094	0.200	0.989	0.999		
R Step Mean	TD	0.176	0.200	0.957	0.602	0.595	0.447
	ASD	0.104	0.200	0.992	1.000		
L Stance CoV	TD	0.202	0.081	0.720	0.000	17.202	0.000
	ASD	0.230	0.024	0.836	0.008		
R Stance CoV	TD	0.301	0.000	0.615	0.000	13.371	0.001
	ASD	0.172	0.200	0.899	0.077		
L Stance Mean	TD	0.192	0.120	0.954	0.562	0.148	0.704
	ASD	0.145	0.200	0.931	0.254		
R Stance Mean	TD	0.165	0.200	0.947	0.450	0.472	0.497
	ASD	0.112	0.200	0.960	0.658		
L Swing CoV	TD	0.225	0.030	0.818	0.005	11.651	0.002
	ASD	0.184	0.153	0.822	0.005		

R Swing CoV	TD	0.245	0.011	0.732	0.000	13.940	0.001
	ASD	0.250	0.009	0.791	0.002		
L Swing Mean	TD	0.121	0.200	0.955	0.572	0.242	0.627
	ASD	0.283	0.001	0.707	0.000		
R Swing Mean	TD	0.160	0.200	0.942	0.372	2.909	0.098
	ASD	0.263	0.004	0.736	0.000		
L Stride CoV	TD	0.279	0.002	0.648	0.000	11.049	0.002
	ASD	0.211	0.055	0.832	0.007		
R Stride CoV	TD	0.309	0.000	0.585	0.000	16.085	0.000
	ASD	0.205	0.070	0.872	0.029		
L Stride Mean	TD	0.174	0.200	0.956	0.593	0.686	0.414
	ASD	0.085	0.200	0.989	0.999		
R Stride Mean	TD	0.167	0.200	0.955	0.577	1.198	0.282
	ASD	0.089	0.200	0.988	0.998		

Table S5

No Relationship between Gait Variability and ADOS Assessment Scores

	Coefficient of Variation for Left Step timing		
Spearman's Rho	Communication (C)	Reciprocal Social Interaction (RSI)	Total (C & RSI)
r	.012	.155	.296
p	.969	.597	.303

	Coefficient of Variation for Right Step timing		
Spearman's Rho	Communication (C)	Reciprocal Social Interaction (RSI)	Total (C & RSI)
r	.311	.244	.314
p	.280	.401	.274

	Coefficient of Variation for Left Stance timing		
Spearman's Rho	Communication (C)	Reciprocal Social Interaction (RSI)	Total (C & RSI)
r	.114	.222	.221
p	.697	.446	.447

	Coefficient of Variation for Right Stance timing		
Spearman's Rho	Communication (C)	Reciprocal Social Interaction (RSI)	Total (C & RSI)
r	.021	.379	.363
p	.944	.181	.202

	Coefficient of Variation for Left Swing timing		

Spearman's Rho	Communication (C)	Reciprocal Social Interaction (RSI)	Total (C & RSI)
r	.064	.228	.199
p	.828	.432	.495

	Coefficient of Variation for Right Swing timing		
Spearman's Rho	Communication (C)	Reciprocal Social Interaction (RSI)	Total (C & RSI)
r	.078	.268	.265
p	.792	.354	.359

	Coefficient of Variation for Left Stride timing		
Spearman's Rho	Communication (C)	Reciprocal Social Interaction (RSI)	Total (C & RSI)
r	.279	.266	.330
p	.335	.358	.250

	Coefficient of Variation for Right Stride timing		
Spearman's Rho	Communication (C)	Reciprocal Social Interaction (RSI)	Total (C & RSI)
r	.037	.326	.274
p	.901	.255	.343

Note. The N = 14 ADOS scores used in this analysis were the ones from Table 2 of supplementary information, consisting of n = 10 clinician scores and n = 4 researcher scores. In the interests of thoroughness, these analyses were repeated (N = 14), giving priority to researcher-rated ADOS scores (n = 13, including research-standard coding of clinical psychologist's ADOS video-recordings n = 9), complemented by

one clinical psychologist-only coded ADOS, where the video recording was not available for researcher-recoding. This resulted in the same conclusions of no relationship between gait timing variability and ADOS.

Table S6

Weight Percentiles of ASD Participants

Participant ID	Age in years	Height (cm)	Weight (kg)	BMI (adults only)	Weight percentiles
2	9.83	126	32	n/a	90
3	11.83	146	42	n/a	76
4	20.83	181	76	23.2	27
11	12.08	147	35	n/a	20
12	14.08	173	54	n/a	31
13	8.42	122	20	n/a	02
14	8.92	139	31	n/a	48
15	10.33	137	28	n/a	18
17	12.08	152	50	n/a	88
18	14.92	175	68	n/a	77
20	29.42	183	63	18.8	06
28	11.25	150	50	n/a	92
29	14.92	170	59	n/a	59
35	20.03	180	108	33.3	83
36	35	192	80	21.7	36
37	8.33	140	35	n/a	82

Note. Weight percentiles used for adults as well as children in order to create weight data that was comparable between children and adults. Both child and adult weight percentile calculations derived from Centers for Disease Control and prevention data (CDC, U.S.): Adult's weight percentiles obtained from https://dqydj.com/weight-percentile-calculator-men-women/.

Child’s percentiles obtained from

https://www.cdc.gov/healthyweight/bmi/calculator.html

Table S7

Test 1 Accuracy Results for Machine Learning Models Performing ASD Classification

Model Type	Accuracy (%)	Mean CV Accuracy (%)
Random Forest	71.43	55.00
Decision Tree	71.43	65.00
Perceptron	28.57	56.67
Support Vector Machine	14.29	58.33
Logistic Regression	14.29	58.33
K-Nearest Neighbour	57.14	48.33
Bagging	71.43	58.33
**AdaBoost**	**85.71**	**45.00**
Gaussian Naïve-Bayes	71.43	51.67
Gradient Boosting	71.43	50.00
Linear Discriminant Analysis	42.86	50.00
Quadratic Discriminant Analysis	14.29	48.33

Table S8

Test 2 Accuracy Results for Machine Learning Models Performing ASD Classification

Model Type	Accuracy (%)	Mean CV Accuracy (%)
Random Forest	71.43	80.00
**Decision Tree**	**85.71**	**76.67**
Perceptron	14.29	58.33
Support Vector Machine	14.29	58.33
Logistic Regression	14.29	58.33
K-Nearest Neighbour	57.14	65.00
Bagging	71.43	83.33
AdaBoost	71.43	66.67
Gaussian Naïve-Bayes	71.43	78.33
Gradient Boosting	71.43	76.67
Linear Discriminant Analysis	71.43	48.33
Quadratic Discriminant Analysis	14.29	55.00

Table S9

Test 3 Accuracy Results for Machine Learning Models Performing ASD Classification

Model Type	Accuracy (%)	Mean CV Accuracy (%)
**Random Forest**	**63.77**	**53.94**
Decision Tree	52.17	53.13
Perceptron	46.38	49.83
Support Vector Machine	44.93	53.52
Logistic Regression	50.72	52.72
K-Nearest Neighbour	57.97	52.47
Bagging	57.97	52.39
AdaBoost	53.62	51.07
Gaussian Naïve-Bayes	52.17	47.99
Gradient Boosting	49.28	52.05
Linear Discriminant Analysis	55.07	49.07
Quadratic Discriminant Analysis	50.72	52.82

Table S10

Test 4 Accuracy Results for Machine Learning Models Performing ASD Classification

Model Type	Accuracy (%)	Mean CV Accuracy (%)
**Random Forest**	**61.80**	**71.87**
Decision Tree	59.55	62.80
Perceptron	40.45	49.81
Support Vector Machine	57.30	70.14
Logistic Regression	48.31	60.02
K-Nearest Neighbour	66.29	70.14
Bagging	64.04	70.15
AdaBoost	53.93	57.79
Gaussian Naïve-Bayes	56.18	59.43
Gradient Boosting	60.67	67.36
Linear Discriminant Analysis	51.69	58.02
Quadratic Discriminant Analysis	60.67	60.62

Table S11

Test 5 Accuracy Results for Machine Learning Models Performing ASD Classification

Model Type	Accuracy (%)	Mean CV Accuracy (%)
**Random Forest**	**75.76**	**68.85**
Decision Tree	54.55	63.08
Perceptron	48.48	50.77
Support Vector Machine	51.52	50.38
Logistic Regression	45.45	52.69
K-Nearest Neighbour	65.15	59.62
Bagging	69.70	67.31
AdaBoost	65.15	61.92
Gaussian Naïve-Bayes	65.15	66.54
Gradient Boosting	69.70	65.77
Linear Discriminant Analysis	63.64	60.00
Quadratic Discriminant Analysis	68.18	68.46

Table S12

Test 6 Accuracy Results for Machine Learning Models Performing ASD Classification

Model Type	Accuracy (%)	Mean CV Accuracy (%)
**Random Forest**	82.18	82.00
Decision Tree	76.24	72.75
Perceptron	53.47	49.50
Support Vector Machine	53.47	50.50
Logistic Regression	43.57	49.50
K-Nearest Neighbour	75.25	73.00
Bagging	84.16	78.00
AdaBoost	70.30	71.75
Gaussian Naïve-Bayes	69.31	67.00
Gradient Boosting	79.21	79.50
Linear Discriminant Analysis	69.31	64.50
Quadratic Discriminant Analysis	78.22	69.50
